# Tremor in multiple sclerosis is associated with cerebello-thalamic pathology

**DOI:** 10.1007/s00702-017-1798-4

**Published:** 2017-11-02

**Authors:** Frederique Boonstra, Grace Florescu, Andrew Evans, Chris Steward, Peter Mitchell, Patricia Desmond, Brad Moffat, Helmut Butzkueven, Scott Kolbe, Anneke van der Walt

**Affiliations:** 1 Department of Anatomy and Neuroscience, University of Melbourne, Melbourne, Australia; 2 Department of Radiology, Royal Melbourne Hospital, University of Melbourne, Melbourne, Australia; 3 Department of Medicine, Royal Melbourne Hospital, Melbourne, Australia; 4 Department of Neurology, The Royal Melbourne Hospital, Level 4 South, 300 Grattan Street, Parkville, VIC 3052 Australia; 5 Melbourne Brain Centre at Royal Melbourne Hospital, Department of Medicine, University of Melbourne, Melbourne, Australia; 6 Multiple Sclerosis Unit, Box Hill Hospital, Box Hill, Australia; 7 Department of Neuroscience, Alfred Central Clinical School, Monash University, Melbourne, Australia

**Keywords:** Multiple sclerosis, Tremor, MRI, Thalamus, Superior cerebellar peduncle

## Abstract

Tremor in people with multiple sclerosis (MS) is a frequent and debilitating symptom with a relatively poorly understood pathophysiology. To determine the relationship between clinical tremor severity and structural magnetic resonance imaging parameters. Eleven patients with clinically definite MS and right-sided upper limb tremor were studied. Tremor severity was assessed using the Bain score (overall severity, writing, and Archimedes spiral drawing). Cerebellar dysfunction was assessed using the Scale for the Assessment and Rating of Ataxia. Dystonia was assessed using the Global Dystonia Scale adapted for upper limb. For all subjects, volume was calculated for the thalamus from T1-weighted volumetric scans using Freesurfer. Superior cerebellar peduncle (SCP) cross-sectional areas were measured manually. The presence of lesions was visually determined and the lesion volumes were calculated by the lesion growth algorithm as implemented in the Lesion Segmentation Toolbox. Right thalamic volume negatively correlated with Bain tremor severity score (*ρ* = − 0.65, *p* = 0.03). Left thalamic volume negatively correlated with general Bain tremor severity score (*ρ* = − 0.65, *p* = 0.03) and the Bain writing score (*ρ* = − 0.65, *p* = 0.03). Right SCP area negatively correlated with Bain writing score (*ρ* = − 0.69, *p* = 0.02). Finally, Bain Archimedes score was significantly higher in patients with lesions in the contralateral thalamus. Whole brain lesion load showed no relationship with tremor severity. These results implicate degeneration of key structures within the cerebello-thalamic pathway as pathological substrates for tremor in MS patients.

## Introduction

Tremor in multiple sclerosis (MS) is a common and disabling symptom. Epidemiological data suggest that tremor affects 26–58% of people with MS and can significantly impact their quality of life (Alusi et al. 2001; Pittock et al. 2004). MS tremor most commonly affects the upper limbs, but has also been observed in the legs, head and trunk (Alusi et al. 1999). MS tremor has postural components and intention components (Koch et al. 2007), and these features can limit patients’ daily functions such that those affected are more likely to be unemployed or retired as a result of their disability (Pittock et al. 2004).

The pathophysiology of MS-related tremor remains poorly understood, as the multifocal nature of MS lesions makes it difficult to identify the precise neuroanatomical substrates of tremor (Feys et al. 2005a, b). Essential tremor (ET) has been more extensively investigated. In ET patients, Contarino et al. (2012) reported a correlation between tremulous movement measured with EEG and fMRI activation in the cerebellum and thalamus. Furthermore, diffusion studies in ET have shown abnormalities in the white matter in the cerebellum, cerebellar peduncles, brainstem, and cerebellar hemispheres. Together, studies on the pathophysiology in ET suggest an important role for the cerebellum and the cerebello-thalamo-cortical pathways (Bucher et al. 1997; Colebatch et al. 1990; Deuschl et al. 2001; Louis et al. 2002; Feys et al. 2005a, b).

Without an understanding of the pathophysiology of MS tremor, development of adequate treatment options is challenging. In MS, cerebellar injury was traditionally thought to be the main pathological driver of MS tremor due to the predominant presence of intention tremor and the correlation between the severity of tremor and dysmetria, dysarthria and dysdiadochokinesia (Diener and Dichgans 1992). However, more recently, careful clinical evaluation of patients with MS tremor has highlighted the clinical commonalities with essential tremor and questioned the relevance of purely cerebellar features on clinical examination (Van der Walt et al. 2012, 2015). These two previous case studies observed the presence of lesions in the cerebellar peduncles (superior and middle) in patients with MS tremor as well as cerebellum (Fahn 1986; Nakamura et al. 1993). Furthermore, animal studies investigating tremor pathogenesis and surgical treatment with deep brain stimulation (DBS) targeting the thalamic nuclei in MS tremor highlight an interaction between the cerebellum and thalamus (Deuschl and Bergman 2002; Koch et al. 2007; Yap et al. 2007). Together, findings in Essential Tremor and MS indicated that tremor in people with MS could result from damage to the cerebello-thalamic connections via the superior cerebellar peduncle (SCP).

For the justification of a future larger study in MS patient with tremor, we conducted a pilot study that aimed to assess the hypothesis that damage to the cerebellar tracts rather than cerebellar hemispheres, specifically the cerebello-thalamic tract, is related to tremor severity in patients with clinically definite MS and unilateral upper limb tremor. We hypothesized that atrophy of structures in the tract (the SCP and thalami) indicative of neuro-axonal degeneration within these structures would correlate with the severity of tremor. This study might provide the necessary support for future larger studies.

## Methods

### Participants

Fourteen patients with clinically definite MS (Polman et al. 2005) and predominantly unilateral MS-related tremor were prospectively identified from the Royal Melbourne Hospital MS Clinic from 2009 to 2010 prior to enrolment in a phase 2 clinical trial examining the efficacy of onabotulinumtoxin-A in MS-related tremor (Van der Walt et al. 2012). Left-hand dominant participants (*n* = 3) were excluded from magnetic resonance imaging (MRI) analysis to focus on lateralization of the tremor pathogenesis in a clearly lateralized group of greatest size. At the time of testing, no patients were on tremor modulating treatment. The project was approved by the Royal Melbourne Hospital Human Research Ethics Committee and informed consent was obtained from each participant prior to participation.

### Clinical assessments

Detailed phenotypical data were collected for each patient, including demographic data, EDSS (Expanded Disability Severity Scale) (Kurtzke 1983), and cerebellar dysfunction scores [Scale for the Assessment and Rating of Ataxia (SARA)] (Schmitz-Hubsch et al. 2006).

A movement disorder neurologist completed a clinical tremor assessment as has been previously described (Van der Walt et al. 2015). Tremor in the affected limb was rated using the Bain score (Bain et al. 1993). The Bain score is a subjective rating scale from 0 to 10 (0 = no tremor and 10 = extremely severe) for overall tremor severity, tremor during handwriting and tremor during drawing of an Archimedes spiral on a pre-drawn pattern. Dystonia (Van der Walt et al. 2015) in the tremor-affected arm was assessed using the Global Dystonia Scale (GDS) in the upper limb only (range 0–10).

### Neuroimaging

**Fig. 1:**
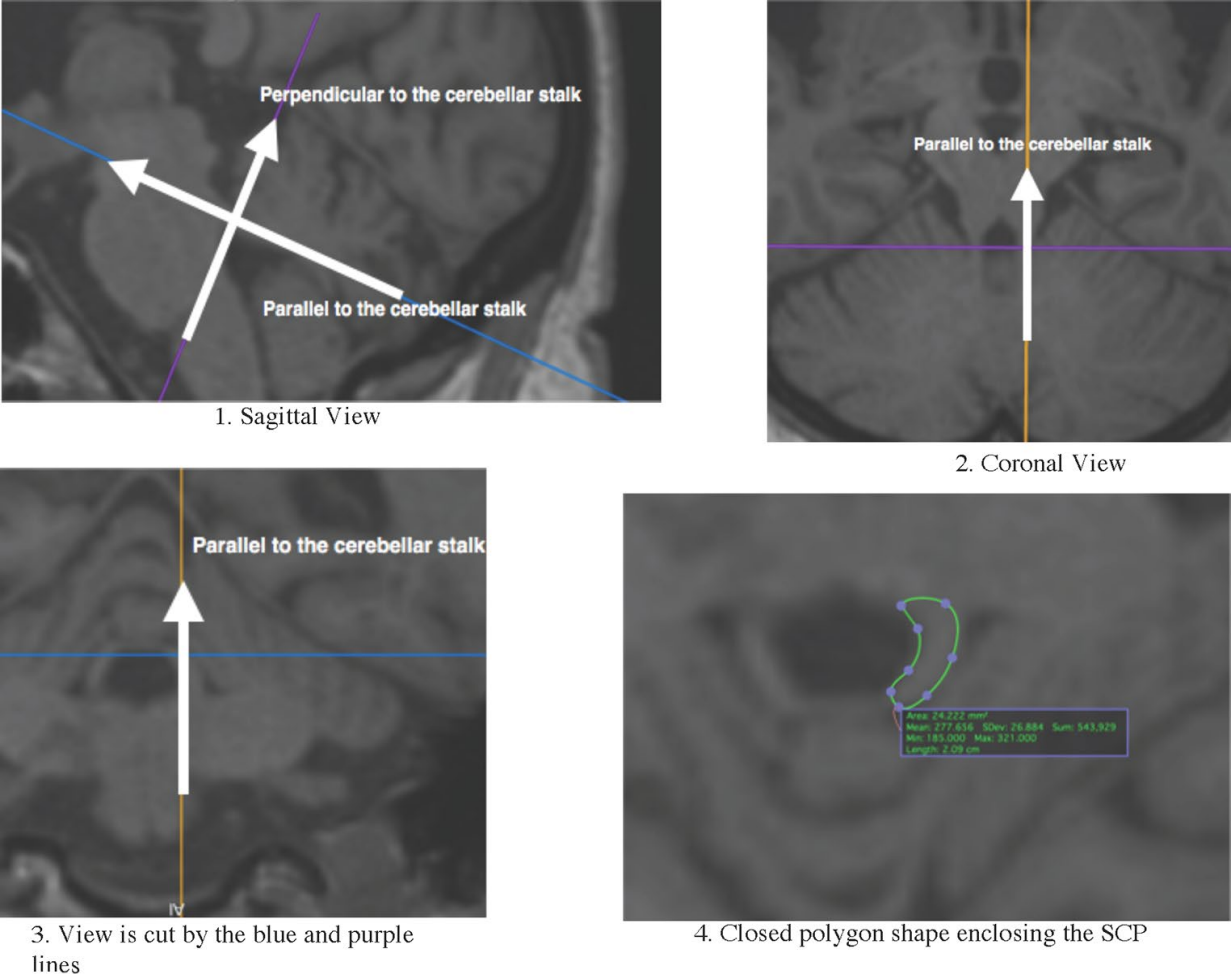
Alignment and manual labelling of the SCP. Step 1 in sagittal view: move the intersection of the axis to the middle of the cerebellar stalk and rotate the blue axis parallel to and the purple axis perpendicular to the cerebellar stalk. Step 2 and step 3: rotate the orange axis parallel to the either left or right cerebellar stalk. Step 4: use a closed polygon shape to map the area of the SCP

Imaging was performed on a 3 Tesla MRI system (Siemens, Erlangen, Germany) and included a 3D MPRAGE T1-weighted scan (TR = 1900, TE = 2.26, TI = 900, FOV = 224 × 256 mm^2^, BW = 200, flip angle = 9, slice thickness = 1 mm and matrix size = 256 × 215 mm) and 3D SPACE FLAIR scan (TR = 6000, TE = 405.0, TI = 2100, FOV = 224 × 256, BW = 700, flip angle = 120, slice thickness = 1 and matrix size = 256 × 222). Regions of interest in the cerebello-thalamic tract included the thalamus and the SCP. T1-weighted MRI scans were processed using Freesurfer (version 5.3) and the standard processing pipeline to obtain volume of the thalamus. The area of the SCP was manually labelled using OsiriX Lite v7.0.4 (Geneva, Switzerland) by two independent raters GF and FB (intraclass correlation = 0.729). The axis in OsiriX were aligned perpendicular and parallel to the cerebellar stalk (see Fig. 1 for a schematic overview of the alignment of the SCP) and then a closed polygon shape was drawn by the raters. The borders of the SCP were defined as by Akhlaghi et al. (2011). Finally, lesions have been segmented by the lesion growth algorithm (Schmidt et al., 2012) as implemented in the LST toolbox version 2.0.15 (http://www.statisticalmodelling.de/lst.html) for SPM. The lesion growth algorithm is able to segment T2-hyperintense lesions from a combination of T1 and FLAIR images. Default settings have been used and this technique have been validated in MS patients (Schmidt et al. 2012). For lesion localization, a neuro-radiologist visually analysed the scans and determined the presence or absence of any lesions within the thalamus and whole cerebellum (including SCP) for each hemisphere.

### Statistical analyses

All statistical analyses were performed using SPSS 23 (IBM). The thalamic volume, SCP area and total lesion load were corrected for intracranial volume (ICV) and age by linear regression. Correlations between tremor scores and corrected imaging parameters were done using non-parametric spearman’s correlation and Mann–Whitney *U* tests as clinical scales are ordinal rather than continual. *p* values below 0.05 were considered significant without correction for multiple comparisons as this is a preliminary exploratory study with low subject numbers.

## Results

Subject demographic and disease data are reported in Table 1. Overall, patients were moderately disabled (EDSS 3–5.5), and had a mild-to-moderate tremor severity (mean Bain tremor severity score 3.21). Half of our patients had a lesion(s) within the ipsilateral and/or contralateral cerebellum/SCP (54.5 and 45.5%, respectively), 27.3% of the patients had a lesion(s) in the ipsilateral thalamus and 18.2% had a lesion(s) in the contralateral thalamus.

**Table 1:** Patient demographics

Cohort (*n* = 11)	
Age, years (mean, SD)	54.1 (± 11.4)
Gender, male (%)	36.4
Disease duration, years (mean, SD)	16.3 (± 8.7)
EDSS score (median, IQR)	4.5 (3–5.5)
SARA score (mean, SD)	11.6 (7.7)
Bain tremor severity score (median, IQR)	3 (2–3)
Bain writing score (median, IQR)	2 (1–4)
Bain Archimedes spiral score (median, IQR)	1 (1–4)
GDS (mean, SD)	3.1 (± 1.2)
ICV, × 10^5^ mm^3^ (mean, SD)	14.5 (± 1.5)
Thalamic volume, right mm^3^ (mean, SD)	6058 (± 916)
Thalamic volume, left mm^3^ (mean, SD)	6865 (± 1267)
SCP area, right mm^2^ (mean, SD)	22.4 (± 3.3)
SCP area, left mm^2^ (mean, SD)	22.4 (± 2.7)
Lesion load, ml (mean, SD)	14.2 (± 10.9)
Lesions in cerebellum/SCP, right (%)	54.5
Lesions in cerebellum/SCP, left (%)	45.5
Lesions in thalamus, right (%)	27.3
Lesions in thalamus, left (%)	18.2

*EDSS* expended disability status score, *SARA* Scale for the Assessment and Rating of Ataxia, *GDS* global dystonia scale (upper limb only), *ICV* intracranial volume, *SCP* superior cerebellar peduncle

Correlation results are summarized in Table 2. There was a significant negative correlation between right thalamic volume and Bain tremor severity score (*ρ* = − 0.647, *p* = 0.031). Left thalamic volume inversely correlated with both the overall Bain tremor severity score (*ρ* = − 0.647, *p* = 0.031) and the Bain writing score (*ρ* = − 0.650, *p* = 0.030). In addition, our data showed a significant negative correlation between right SCP area and Bain Writing score (*ρ* = − 0.693, *p* = 0.018). Finally, loss of volume in the left SCP area was associated with a higher upper limb GDS (*ρ* = − 0.697, *p* = 0.017). There were no significant correlations between whole brain lesion load and any of the tremor severity scores; however, patients with lesion(s) within the contralateral thalamus had an increased Bain Archimedes score (*U* = 0.5, *p* = 0.036) compared to patients without lesion(s) within the contralateral thalamus. None of the imaging parameters correlated with the SARA score.

**Table 2:** Correlation between tremor and dystonia severity scores and MRI measures

	Bain tremor severity score (*ρ* value, *p* value)	Bain writing score (*ρ* value, *p* value)	Bain Archimedes spiral score (*ρ* value, *p* value)	SARA score (*ρ* value, *p* value	GDS (*ρ* value, *p* value)
Thalamic volume, right	*ρ* **=** **−** **0.647** ***p*** **=** 0.031*	*ρ* = − 0.593 *p* = 0.055	*ρ* = − 0.507 *p* = 0.112	*ρ* = − 0.427 *p* = 0.190	*ρ* = − 0.524 *p* = 0.098
Thalamic volume, left	*ρ* **=** **−** **0.647** ***p*** **=** 0.031*	*ρ* **=** **−** **0.650** ***p*** **=** 0.030*	*ρ* = − 0.507 *p* = 0.112	*ρ* = − 0.309 *p* = 0.255	*ρ* = − 0.557 *p* = 0.075*
SCP area, right	*ρ* = − 0.338 *p* = 0.309	*ρ* **=** **−** **0.693** ***p*** **=** 0.018*	*ρ* = − 0.578 *p* = 0.062	*ρ* = − 0.154 *p* = 0.670	*ρ* = − 0.509 *p* = 0.110
SCP area, left	*ρ* = − 0.162 *p* = 0.635	*ρ* = − 0.497 *p* = 0.120	*ρ* = − 0.382 *p* = 0.246	*ρ* = 0.191 *p* = 0.574	*ρ* **=** **−** **0.697** ***p*** **=** 0.017*
Lesions					
Whole brain (volume)	*ρ* = 0.206 *p* = 0.543	*ρ* = 0.363 *p* = 0.272	*ρ* = 0.406 *p* = 0.215	*ρ* = − 0.136 *p* = 0.689	*ρ* = 0.106 *p* = 0.757
Cerebellar/SCP, right (presence)	*U* = 13.5 *p* = 0.792	*U* = 6.0 *p* = 0.126	*U* = 7.5 *p* = 0.177	*U* = 12.0 *p* = 0.662	*U* = 11.0 *p* = 0.537
Cerebellar/SCP, left (presence)	*U* = 15.0 *p* = 1.00	*U* = 14.0 *p* = 0.931	*U* = 12.5 *p* = 0.662	*U* = 9.0 *p* = 0.329	*U* = 14.5 *p* = 0.931
Thalamus, right (presence)	*U* = 9.0 *p* = 0.630	*U* = 10.0 *p* = 0.776	*U* = 7.0 *p* = 0.376	*U* = 11.0 *p* = 0.921	*U* = 8.0 *p* = 0.497
Thalamus, left (presence)	*U* = 3.0 *p* = 0.218*	*U* = 2.0 *p* = 0.145	***U*** **=** **0.5** ***p*** **=** 0.036*	*U* = 9.0 *p* = 1.00	*U* = 2.0 *p* = 0.145

*SARA* Scale for the Assessment and Rating of Ataxia, *GDS* Global Dystonia Scale (upper limb only), *SCP* superior cerebellar peduncle, *ρ* Spearman rho, *U* Mann–Whitney *U* test

Asterisk and bold text indicates significance *p* < 0.05

## Discussion

This study investigated the involvement of the cerebello-thalamic tract in the pathogenesis of unilateral MS upper limb tremor. The thalamus was of particular interest as thalamic atrophy had been correlated with worse prognosis in MS patients (Rocca et al. 2010), is present in the very early stages of MS (Azevedo et al. 2015; Bergsland et al. 2012), and has been shown to allow differentiation from other MRI white matter pathologies (Solomon et al. 2017). We found that contra- and ipsilateral thalamic volumes and ipsilateral SCP area inversely correlated with increased unilateral tremor severity scores. These regions of volume loss are in line with the predicted neuroanatomy of the cerebello-thalamic tract. The lack of significant correlation between neuroimaging parameters and cerebellar dysfunction measured by the SARA score, likely reflects the effects of gait and unaffected upper limb scores when calculating the total SARA score.

Our findings are consistent with involvement of the cerebello-thalamic tract in MS tremor pathogenesis. Traditional views of MS tremor emphasize a central role for the cerebellum in MS tremor due to the predominant presence of intention tremor and the correlation between the severity of tremor and dysmetria, dysarthria and dysdiadochokinesia. However, animal studies of tremor pathogenesis and surgical treatment with deep brain stimulation (DBS) targeting the thalamic nuclei in MS tremor highlight an interaction between the cerebellum, cortex, basal ganglia and thalamus (Deuschl and Bergman 2002; Koch et al. 2007; Yap et al. 2007). Clinical neuroimaging studies of MS tremor are, however, rare. Two case reports have both associated an acute SCP demyelinating lesion with acute tremor onset (Fahn 1986; Nakamura et al. 1993). Combined with a positive clinical trial with onabotulinumtoxin-A, a treatment typically used in focal dystonias (Van der Walt et al. 2012), these studies provide further support for tract-based pathogenesis of tremor. Finally, Feys et al. (2005a, b) demonstrated a link between MS tremor severity and T2 lesion load in the contralateral pons but not with the lesion load in the cerebellum.

We found no evidence of a correlation between the tremor severity scores and the overall lesion load in the brain in mild-to-moderate unilateral MS tremor. Interestingly, the presence of lesions within in contralateral thalamus correlated to an increase in tremor severity in unilateral MS tremor. These findings support the lateralization and localization of tremor pathophysiology. Consistent with this, Feys et al. (2005a, b) studied the relationship between lesion load and tremor severity in a significantly disabled cohort (EDSS from 6 to 8) where 93% of participants had bilateral tremor. The authors found no correlation between overall lesion load and tremor severity; however, they showed the importance of lesion topography and tremor severity. Specifically, they found a correlation between lesion load in the contralateral pons and tremor severity. Furthermore, the correlation was related to the degree of bilateral tremor. The pons is known to contains nuclei that are involved in a variety of functions that are commonly symptomatic in MS, including pyramidal function, bladder control, equilibrium and facial sensation (Brodal 2014; Saladin Kenneth 2007) and a correlation between pontine lesion load and severe bilateral tremor scores are therefore not surprising. Here, we further highlight the importance of lesion topography and further study into lesion topography and tremor severity in unilateral tremor is warranted.

Our findings were demonstrated in a small sample size limiting our ability to correct for EDSS, disease duration and other factors. Furthermore, the lack of controls and cross-sectional nature of the study hinders any cause-and-effect analyses. Despite these limitations, this study is hypothesis generating in an area that is currently poorly understood.

In conclusion, we have found significant correlations between functional measures of tremor and brain abnormalities as indicated by MRI. The data support the feasibility of further, larger studies using more advanced imaging techniques (e.g., tractography, DWI functional MRI) to better define tremor pathogenesis in MS.

## References

[CR1] Akhlaghi H, Corben L, Georgiou-Karistianis N, Bradshaw J, Storey E, Delatycki MB, Egan GF (2011). Superior cerebellar peduncle atrophy in Friedreich’s ataxia correlates with disease symptoms. Cerebellum.

[CR2] Alusi SH, Glickman S, Aziz TZ, Bain PG (1999). Tremor in multiple sclerosis. J Neurol Neurosurg Psychiatry.

[CR3] Alusi SH, Worthington J, Glickman S, Bain PG (2001). A study of tremor in multiple sclerosis. Brain.

[CR4] Azevedo CJ, Overton E, Khadka S, Buckley J, Liu S, Sampat M, Pelletier D (2015). Early CNS neurodegeneration in radiologically isolated syndrome. Neurol Neuroimmunol Neuroinflammation.

[CR5] Bain PG, Findley LJ, Atchison P, Behari M, Vidailhet M, Gresty M, Marsden CD (1993). Assessing tremor severity. J Neurol Neurosurg Psychiatry.

[CR6] Bergsland N, Horakova D, Dwyer MG, Dolezal O, Seidl ZK, Vaneckova M, Zivadinov R (2012). Subcortical and cortical gray matter atrophy in a large sample of patients with clinically isolated syndrome and early relapsing-remitting multiple sclerosis. AJNR Am J Neuroradiol.

[CR7] Brodal P (2014). Pontine nuclei encyclopedia of the neurological sciences.

[CR8] Bucher SF, Seelos KC, Dodel RC, Reiser M, Oertel WH (1997). Activation mapping in essential tremor with functional magnetic resonance imaging. Ann Neurol.

[CR9] Fahn S (1986) What is it? Case 1 1986. Presentation of a case. Mov Disord 1(4):275–280. 10.1002/mds.870010408

[CR10] Colebatch JG, Findley LJ, Frackowiak RS, Marsden CD, Brooks DJ (1990). Preliminary report: activation of the cerebellum in essential tremor. Lancet.

[CR11] Contarino MF, Groot PFC, van der Meer JN, Bour LJ, Speelman JD, Nederveen AJ, van Rootselaar A-F (2012). Is there a role for combined EMG-fMRI in exploring the pathophysiology of essential tremor and improving functional neurosurgery?. PLoS One.

[CR12] Deuschl G, Bergman H (2002). Pathophysiology of nonparkinsonian tremors. Mov Disord.

[CR13] Deuschl G, Raethjen J, Lindemann M, Krack P (2001). The pathophysiology of tremor. Muscle Nerve.

[CR14] Diener HC, Dichgans J (1992). Pathophysiology of cerebellar ataxia. Mov Disord.

[CR15] Feys P, Helsen W, Liu X, Mooren D, Albrecht H, Nuttin B, Ketelaer P (2005). Effects of peripheral cooling on intention tremor in multiple sclerosis. J Neurol Neurosurg Psychiatry.

[CR16] Feys P, Maes F, Nuttin B, Helsen W, Malfait V, Nagels G, Liu X (2005). Relationship between multiple sclerosis intention tremor severity and lesion load in the brainstem. NeuroReport.

[CR17] Koch M, Mostert J, Heersema D, De Keyser J (2007). Tremor in multiple sclerosis. J Neurol.

[CR18] Kurtzke JF (1983). Rating neurologic impairment in multiple sclerosis: an expanded disability status scale (EDSS). Neurology.

[CR19] Louis ED, Shungu DC, Chan S, Mao X, Jurewicz EC, Watner D (2002). Metabolic abnormality in the cerebellum in patients with essential tremor: a proton magnetic resonance spectroscopic imaging study. Neurosci Lett.

[CR20] Nakamura R, Kamakura K, Tadano Y, Hosoda Y, Nagata N, Tsuchiya K, Shibasaki H (1993). MR imaging findings of tremors associated with lesions in cerebellar outflow tracts: report of two cases. Mov Disord.

[CR21] Pittock SJ, McClelland RL, Mayr WT, Rodriguez M, Matsumoto JY (2004). Prevalence of tremor in multiple sclerosis and associated disability in the Olmsted County population. Mov Disord.

[CR22] Polman CH, Reingold SC, Edan G, Filippi M, Hartung HP, Kappos L, Wolinsky JS (2005). Diagnostic criteria for multiple sclerosis: 2005 revisions to the “McDonald Criteria”. Ann Neurol.

[CR24] Rocca MA, Mesaros S, Pagani E, Sormani MP, Comi G, Filippi M (2010). Thalamic damage and long-term progression of disability in multiple sclerosis. Radiology.

[CR25] Saladin Kenneth S (2007). Anatomy and physiology the unity of form and function.

[CR26] Schmidt P, Gaser C, Arsic M, Buck D, Forschler A, Berthele A, Muhlau M (2012). An automated tool for detection of FLAIR-hyperintense white-matter lesions in Multiple Sclerosis. NeuroImage.

[CR27] Schmitz-Hubsch T, du Montcel ST, Baliko L, Berciano J, Boesch S, Depondt C, Fancellu R (2006). Scale for the assessment and rating of ataxia: development of a new clinical scale. Neurology.

[CR28] Solomon AJ, Watts R, Dewey BE, Reich DS (2017). MRI evaluation of thalamic volume differentiates MS from common mimics. Neurol Neuroimmunol Neuroinflammation.

[CR29] Van der Walt A, Sung S, Spelman T, Marriott M, Kolbe S, Mitchell P, Butzkueven H (2012). A double-blind, randomized, controlled study of botulinum toxin type A in MS-related tremor. Neurology.

[CR30] Van der Walt A, Buzzard K, Sung S, Spelman T, Kolbe SC, Marriott M, Evans A (2015). The occurrence of dystonia in upper-limb multiple sclerosis tremor. Mult Scler.

[CR31] Yap L, Kouyialis A, Varma TR (2007). Stereotactic neurosurgery for disabling tremor in multiple sclerosis: thalamotomy or deep brain stimulation?. Br J Neurosurg.

